# Association between vitamin D supplementation and mortality in elderly critically ill patients: a retrospective propensity score–matched cohort study

**DOI:** 10.3389/fnut.2026.1932001

**Published:** 2026-09-17

**Authors:** Zhongmin Tian, Yanjun Liu, Yijing Zhou, Lu Wang, Yeqiong He, Lei Zuo, Jixin Chen, Fan Wang, Haitao Liu

**Affiliations:** 1 Departments of Critical Care Medicine, Harbin Medical University Cancer Hospital, Harbin Medical University, Harbin, Heilongjiang, China; 2 Department of Epidemiology, School of Public Health, Harbin Medical University, Harbin, Heilongjiang, China

**Keywords:** elderly patients, intensive care, mortality, prognosis, vitamin D supplementation

## Abstract

**Purpose:**

Vitamin D deficiency is highly prevalent among elderly critically ill patients and has been associated with adverse outcomes. However, evidence regarding the prognostic benefit of vitamin D supplementation in this population remains limited. This study aimed to investigate the association between vitamin D supplementation and 28-day mortality in elderly patients admitted to the intensive care unit (ICU).

**Methods:**

This retrospective cohort study utilized data from the medical information mart for intensive care IV (MIMIC-IV) database, including patients aged ≥65 years. Propensity score matching (PSM) balanced baseline characteristics between patients who received vitamin D within 48 h of ICU admission and those who did not. Cox proportional hazards models estimated hazard ratios (HRs) with 95% confidence intervals (CIs), and E-value analysis assessed unmeasured confounding.

**Results:**

After PSM, 8,111 patients were included, with 1,338 (16.5%) deaths within 28 days. The 28-day mortality rate was significantly lower in the vitamin D supplementation group than in the non-supplemented group (14.5% vs. 17.0%, *P* = 0.017). Multivariable Cox regression demonstrated that vitamin D supplementation was independently associated with lower 28-day mortality (HR = 0.819, 95% CI: 0.710–0.946, *P* = 0.006). Subgroup analyses revealed more pronounced associations in females and patients without certain chronic comorbidities.

**Conclusion:**

Vitamin D supplementation within 48 h of ICU admission was significantly associated with reduced 28-day mortality in elderly critically ill patients. Further prospective interventional studies are required to verify causal relationships and optimize administration regimens.

## 1 Introduction

With the rapid global population aging, the proportion of elderly patients admitted to intensive care units (ICU) continues to increase (1, 2). Patients aged 65 years and older account for approximately half of all ICU admissions, and those aged 80 years and above represent about 10% (3). Due to immunosenescence and reduced multiorgan reserve, elderly patients have the highest risk of ICU mortality. Studies have shown that compared with younger patients, elderly critically ill individuals experience a higher rate of adverse outcomes (1, 4), with the overall prevalence of multidimensional frailty (MPI-3) reaching 26.8% (5). They often present with more severe cognitive impairment (6, 7), multiple comorbidities, a higher risk of depression and malnutrition. These factors contribute to longer ICU stays, higher readmission rates, greater consumption of medical resources (such as mechanical ventilation and renal replacement therapy), and ultimately higher mortality (8, 9). Although advances in organ support technologies have greatly improved critical care, outcomes for elderly patients remain unsatisfactory (10), which may be related to widespread metabolic and nutritional disorders. Previous studies reported that up to 90% of elderly ICU patients suffer from malnutrition, accompanied by significant muscle loss and resistance to protein synthesis (11, 12). Therefore, precision nutritional interventions may be a potential strategy to improve outcomes in this population (13–15).

Among various nutrients, vitamin D has garnered increasing attention for its dual roles in muscle synthesis and immune regulation. Vitamin D is a fat-soluble vitamin derived from both endogenous synthesis in the skin under ultraviolet (UV) radiation and exogenous intake from foods such as fish, eggs, milk, and animal liver (16). 25-hydroxyvitamin D_3_ is the major circulating form and the most reliable biomarker of vitamin D status. Its active form, 1,25-dihydroxyvitamin D_3_, exerts biological effects by binding to vitamin D receptors widely distributed in the myocardium, vascular smooth muscle, brain, liver, kidney, bone, parathyroid gland, and various immune cells (17, 18). In addition to its classical role in regulating calcium–phosphorus homeostasis and maintaining skeletal health, vitamin D also modulates immune responses, cardiovascular function, and cellular proliferation and differentiation (19, 20). These pleiotropic physiological effects suggest that vitamin D may exert an important influence on clinical outcomes in critically ill patients.

Although several meta-analyses of RCTs have suggested that vitamin D supplementation may be associated with reduced short-term mortality in critically ill patients (RR = 0.83, 95% CI: 0.70–0.98) (21, 22), the evidence remains inconsistent. Some meta-analyses focusing on high-dose vitamin D3 supplementation failed to demonstrate a significant mortality benefit at 28 days (RR = 0.93, 95% CI: 0.78–1.11) or 90 days (23). Furthermore, a recent narrative review of RCTs published in the last five years concluded that while vitamin D supplementation appears safe and may reduce ICU stay duration and mechanical ventilation time, its impact on overall mortality remains uncertain (24). A large-scale RCT also failed to demonstrate prognostic benefits of vitamin D for critically ill patients (25). These discrepancies may be attributable to differences in baseline vitamin D status, supplementation dose, route of administration, timing of intervention, and patient heterogeneity (24). However, the benefit of vitamin D supplementation in elderly critically ill patients remain uncertain.

Despite growing interest, robust large-scale evidence on whether vitamin D supplementation improves outcomes in elderly critically ill patients remains limited. Therefore, this retrospective cohort study was conducted using the MIMIC-IV database to evaluate the association between vitamin D supplementation within 48 h of ICU admission and 28-day mortality in elderly critically ill patients, with the aim of providing evidence for individualized nutritional strategies in this population.

## 2 Methods

### 2.1 Data source

The medical information mart for intensive care IV (MIMIC-IV) is a large, publicly available electronic health record (EHR) database. It contains de-identified clinical data of more than 90,000 ICU admissions from the Beth Israel Deaconess Medical Center (BIDMC) in Boston, USA, between 2008 and 2022 (26). The use of the MIMIC-IV database was approved by the Institutional Review Boards of BIDMC and Massachusetts Institute of Technology (MIT). Because all data are de-identified and publicly available, the requirement for informed consent was waived. One author (TZM) obtained database access through the Collaborative Institutional Training Initiative (CITI) certification (ID: 60189133). The study was conducted in accordance with the strengthening the reporting of observational studies in epidemiology (STROBE) guidelines.

### 2.2 Study population

An age threshold of ≥65 years was selected to define elderly patients, in accordance with the widely accepted international definition of older adults adopted by the World Health Organization and the United Nations, as well as the established literature in critical care geriatric research (27). This cutoff also aligns with the age standard used in the MIMIC-IV database for geriatric subgroup analyses and facilitates comparison with prior large-scale observational studies on elderly ICU populations, which have consistently employed the same threshold (28). Patients with an ICU length of stay of < 24 h were excluded to ensure adequate observation time and data integrity.

### 2.3 Exposure and outcome

The exposure of interest was vitamin D supplementation within 48 h after ICU admission. Vitamin D supplementation was identified from medication administration records. Vitamin D supplementation was identified from medication administration records in the MIMIC-IV database, and vitamin D preparations were classified according to the generic sequence number (GSN), generic drug name, and formulation specifications. The included vitamin D medications corresponded to GSN codes 002169, 002171, 002173, 002174, 019166, and 028465. Specifically, GSN 002169 and 002171 correspond to ergocalciferol (vitamin D_2_), while GSN 019166, 002173, 002174, and 028465 correspond to cholecalciferol (vitamin D_3_). Thus, all included formulations were nutritional-type vitamin D preparations; calcitriol and other active vitamin D analogs were excluded. The predominant formulations were 400 IU or 1,000 IU tablets, administered orally or via nasogastric tube, with a minority of patients receiving capsules or liquid preparations. The cumulative dose within 48 h of ICU admission ranged from 400 to 4,000 IU, with a median cumulative dose of 2,000 IU (IQR: 1,600–3,600 IU). All vitamin D administrations were enteral; no intravenous or intramuscular routes were recorded. Structured records did not provide specific clinical indications linked to each vitamin D order; therefore, we did not classify the indications for use.

The primary outcome was 28-day all-cause mortality, defined as death from any cause within 28 days after ICU admission. Mortality data were obtained from hospital records and verified using follow-up information after discharge.

### 2.4 Data extraction

All data were extracted using structured query language (SQL) in a database server environment. The SQL scripts are publicly available on GitHub. Demographic variables (age, sex), clinical outcomes (28-day mortality, ICU mortality, ICU length of stay), and treatment-related variables within the first 24 h after ICU admission were collected. Treatments included invasive mechanical ventilation, continuous renal replacement therapy (CRRT), and the use of vasoactive agents (dopamine, dobutamine, epinephrine, norepinephrine).

Comorbidities were defined using the charlson comorbidity index (CCI) and included renal disease, diabetes, chronic pulmonary disease, cerebrovascular disease, peripheral vascular disease, congestive heart failure, myocardial infarction, severe liver disease, and malignancy. Disease severity was assessed using the sequential organ failure assessment (SOFA) and the simplified acute physiology score II (SAPS II) at ICU admission. Vital signs (heart rate, blood pressure, respiratory rate, temperature, oxygen saturation) and laboratory variables (blood glucose, red and white blood cell counts, platelet count, electrolytes, bicarbonate, blood urea nitrogen, and creatinine) were collected within 24 h after ICU admission.

### 2.5 Statistical analysis

Variables with more than 20% missing data were excluded. Multiple imputation was used to estimate missing values for remaining variables. Variance inflation factors (VIFs) were calculated to detect multicollinearity, with VIF < 5 indicating no significant collinearity.

Patients were divided into two groups according to vitamin D supplementation status. Continuous variables were expressed as medians (interquartile ranges) and compared using the Kruskal–Wallis test. Categorical variables were presented as numbers (percentages) and compared using the chi-square test or Fisher's exact test as appropriate.

Propensity score matching (PSM) was performed to minimize baseline confounding. Propensity scores were estimated using a logistic regression model including age, sex, CCI, SOFA score, and SAPS II score. Nearest-neighbor matching was performed in a 1:4 ratio with a caliper width of 0.02. Covariate balance was assessed using standardized mean differences (SMDs), with SMD < 0.10 indicating acceptable balance.

To evaluate the association between vitamin D supplementation and mortality, Kaplan–Meier curves were plotted to compare 28-day survival between groups using the log-rank test. Cox proportional hazards models were applied to estimate hazard ratios (HRs) and 95% confidence intervals (CIs) for 28-day all-cause mortality. Three models were constructed: Model 1 (unadjusted), Model 2 (adjusted for age, sex, CCI, SOFA, SAPS II), and Model 3 (fully adjusted for demographic factors, comorbidities, vital signs, laboratory tests, and treatment variables). The proportional hazards assumption was evaluated using Schoenfeld residuals. We assessed the proportional hazards assumption of the Cox model using tests based on scaled Schoenfeld residuals, including both individual tests for each covariate in the model and a global test for the model as a whole. A *P*-value > 0.05 was considered to indicate no evidence of violation of the proportional hazards assumption. For covariates that violated the proportional hazards assumption, we further constructed extended Cox models by incorporating interaction terms between the respective covariates and log(1+time), allowing their regression coefficients to vary over follow-up time. The fit of the original Cox models was compared with that of the corresponding extended Cox models using likelihood ratio tests.

Subgroup analyses were conducted according to sex and major comorbidities. For sensitivity analysis, the E-value was calculated to assess the robustness of the findings to potential unmeasured confounding. All tests were two-sided, and a *P*-value < 0.05 was considered statistically significant. Statistical analyses were performed using R software (version 4.4.0).

To further evaluate the association between vitamin D administration characteristics and 28-day ICU mortality risk, we performed a 48-h landmark analysis restricted to patients who received vitamin D supplementation. Patients who died within the first 48 h after ICU admission were excluded, and follow-up was calculated from 48 h after ICU admission until death or day 28. Cumulative vitamin D dose was calculated based on medication records within the first 48 h after ICU admission. Six patients were excluded from the cumulative dose analysis because key information on the number of vitamin D administrations within the first 48 h was missing, to ensure completeness of exposure records. Time to first administration was defined as the time from ICU admission to the first recorded vitamin D administration and was converted from days to hours; patients with missing time to first administration were excluded from the corresponding analysis. Restricted cubic spline Cox proportional hazards regression models with four knots were used to evaluate the overall association and potential non-linear relationships between cumulative vitamin D dose and time to first administration and 28-day ICU mortality risk. Knots were placed at the 5th, 35th, 65th, and 95th percentiles of the corresponding exposure distribution, and the median cumulative dose (2,000 IU) and median time to first administration (5.8 h) were used as reference values, respectively. The models were adjusted for sex, age, Charlson comorbidity index, severe liver disease, malignant cancer, renal disease, diabetes, chronic pulmonary disease, cerebrovascular disease, peripheral vascular disease, congestive heart failure, myocardial infarction, SOFA score, SAPS II score, vital signs, laboratory parameters, invasive mechanical ventilation, continuous renal replacement therapy, and vasoactive agent use. Wald tests were used to evaluate the overall association and non-linear components for each exposure variable with 28-day ICU mortality risk.

## 3 Results

### 3.1 Baseline characteristics

A total of 28,691 critically ill patients were included in the cohort. Among them, 1,627 patients received vitamin D supplementation within 48 h after ICU admission. After propensity score matching, 8,111 patients were included in the matched cohort. The patient selection process is shown in Figure 1. We further analyzed the baseline characteristics of the two patient groups (Table 1). Regarding physiological parameters, there were significant group differences in systolic blood pressure, respiratory rate, body temperature, and oxygen saturation. Laboratory results showed that the supplementation group had lower white blood cell counts (SMD = 0.132) and higher platelet counts (SMD = 0.161). Renal function indicators, including serum creatinine (SMD = 0.201) and blood urea nitrogen (SMD = 0.223), were notably higher. In terms of comorbidities, the supplementation group had a higher prevalence of diabetes (38.0% vs. 32.6%, SMD = 0.114) and chronic kidney disease (32.1% vs. 24.4%, SMD = 0.172), but a lower rate of invasive mechanical ventilation (21.5% vs. 35.9%, SMD = 0.323). After propensity score matching, baseline characteristics were well balanced between the two groups. Most covariates achieved satisfactory balance with standardized mean differences (SMD) < 0.10, including demographic variables (age, sex), vital signs (heart rate, blood pressure), laboratory tests (glucose, electrolytes), clinical severity scores (Charlson Comorbidity Index, SOFA, SAPS II), and most comorbidities and ICU interventions. Minor residual imbalances (SMD ≥ 0.10) were observed for oxygen saturation (SMD = 0.112), renal indicators (BUN: SMD = 0.183; creatinine: SMD = 0.179), certain comorbidities (renal disease: SMD = 0.122; cerebrovascular disease: SMD = 0.118; congestive heart failure: SMD = 0.121), and invasive ventilation (SMD = 0.190). Overall, the two groups were highly comparable after matching.

**Figure 1:**
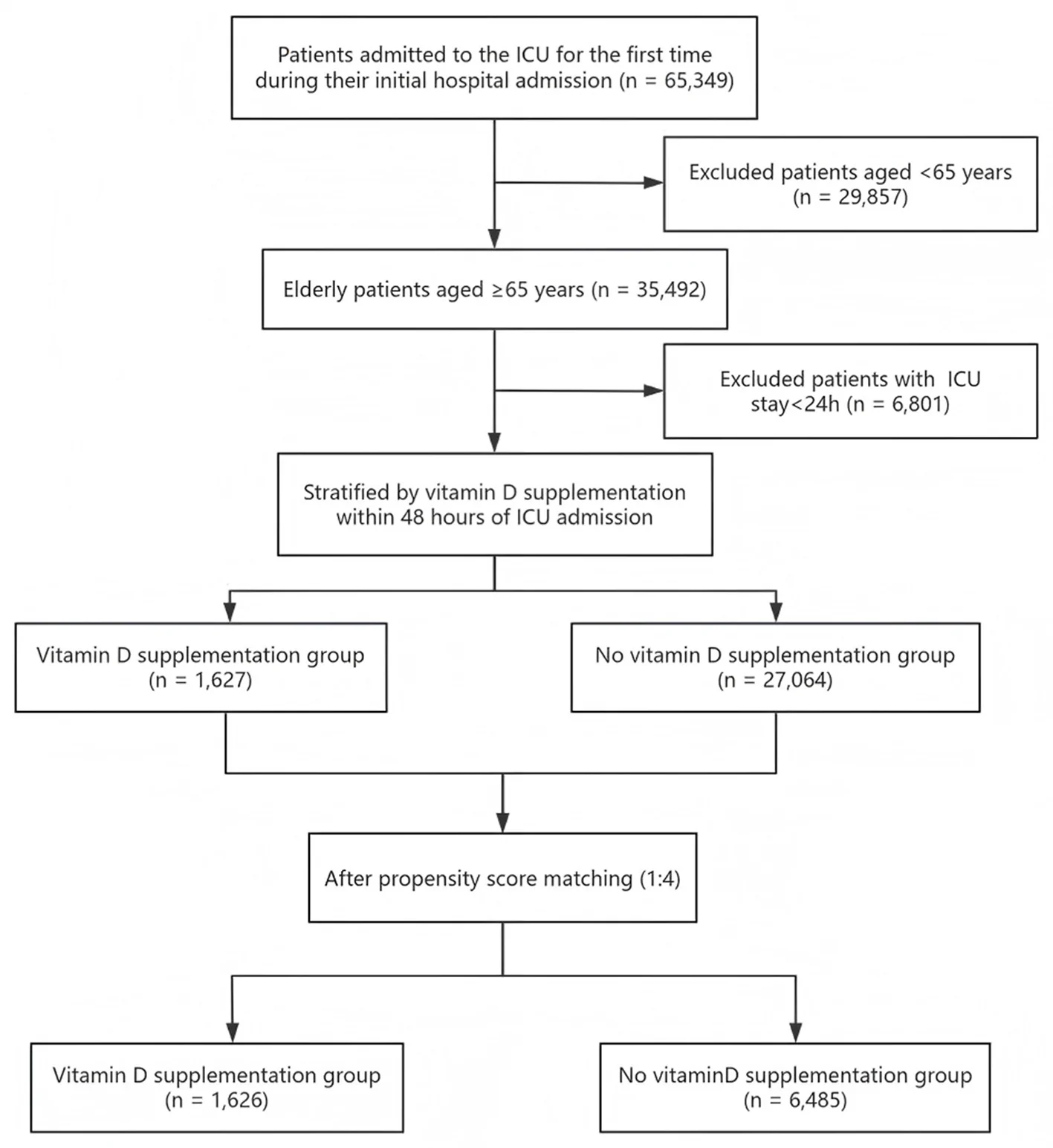
Patient selection flow diagram for the study of vitamin D supplementation in elderly ICU patients.

**Table 1:** Baseline table of the study population.

Variable	Non-supplementation	Vitamin D supplementation	*P*-value	SMD
Age, median [IQR], years	76.6 [70.6, 83.5]	78.9 [72.1, 85.7]	< 0.001	0.211
Male sex, *n* (%)	14,938 (55.2)	711 (43.7)	< 0.001	0.231
Heart rate, median [IQR], bpm	82.0 [73.0, 96.0]	82.0 [72.0, 96.0]	0.27	0.041
Systolic BP, median [IQR], mmHg	123.0 [107.0, 141.0]	125.0 [109.0, 144.0]	< 0.001	0.1
Diastolic BP, median [IQR], mmHg	64.0 [54.0, 76.0]	65.0 [55.0, 77.0]	0.044	0.04
Respiratory rate, breaths/min	18.0 [15.0, 22.0]	19.0 [15.0, 23.0]	< 0.001	0.099
Body temperature, °C	36.6 [36.3, 36.9]	36.7 [36.4, 36.9]	< 0.001	0.11
Oxygen saturation, %	98.0 [95.0, 100.0]	97.0 [95.0, 100.0]	< 0.001	0.132
Glucose level, mg/dL	134.0 [109.0, 170.0]	129.0 [104.0, 168.0]	< 0.001	0.015
WBC count, × 10^9^/L	10.8 [7.9, 14.7]	9.9 [7.3, 13.5]	< 0.001	0.06
Platelet count, × 10^9^/L	182.0 [134.0, 244.0]	196.0 [145.0, 253.0]	< 0.001	0.079
Sodium, mmol/L	138.0 [135.0, 141.0]	138.0 [135.5, 141.0]	0.013	0.012
Potassium, mmol/L	4.2 [3.8, 4.7]	4.2 [3.8, 4.6]	0.761	0.003
Bicarbonate, mmol/L	23.0 [20.0, 25.0]	23.0 [21.0, 25.0]	0.002	0.062
BUN, mg/dL	20.0 [15.0, 32.0]	22.0 [15.0, 36.0]	< 0.001	0.141
Creatinine, mg/dL	1.0 [0.8, 1.4]	1.0 [0.8, 1.6]	< 0.001	0.106
Charlson comorbidity index	6.0 [4.0, 8.0]	6.0 [5.0, 8.0]	< 0.001	0.193
SOFA score	2.0 [1.0, 4.0]	1.0 [0.0, 3.0]	< 0.001	0.207
SAPS II score	38.0 [32.0, 47.0]	37.0 [31.0, 45.0]	< 0.001	0.115
Chronic pulmonary disease, *n* (%)	7,143 (26.4)	481 (29.6)	0.006	0.071
Diabetes, *n* (%)	8,828 (32.6)	619 (38.0)	< 0.001	0.114
Renal disease, *n* (%)	6,612 (24.4)	523 (32.1)	< 0.001	0.172
Congestive heart failure, *n* (%)	8,933 (33.0)	684 (42.0)	< 0.001	0.187
ICU mortality, *n* (%)	2,378 (8.8)	100 (6.1)	< 0.001	0.101
In-hospital mortality, *n* (%)	3,557 (13.1)	165 (10.1)	< 0.001	0.094

Data are presented as median [IQR] or *n* (%). SMD, standardized mean difference; BUN, blood urea nitrogen; SOFA, sequential organ failure assessment.

### 3.2 Association between vitamin D supplementation and all-cause mortality

In the propensity score–matched cohort, the 28-day all-cause mortality was 14.5% (236/1626) in the vitamin D group and 17.0% (1102/6485) in the non-supplementation group. The Kaplan–Meier survival curves (Figure 2) showed that patients receiving vitamin D supplementation had better survival during the 28-day follow-up period (log-rank *P* < 0.05).

**Figure 2:**
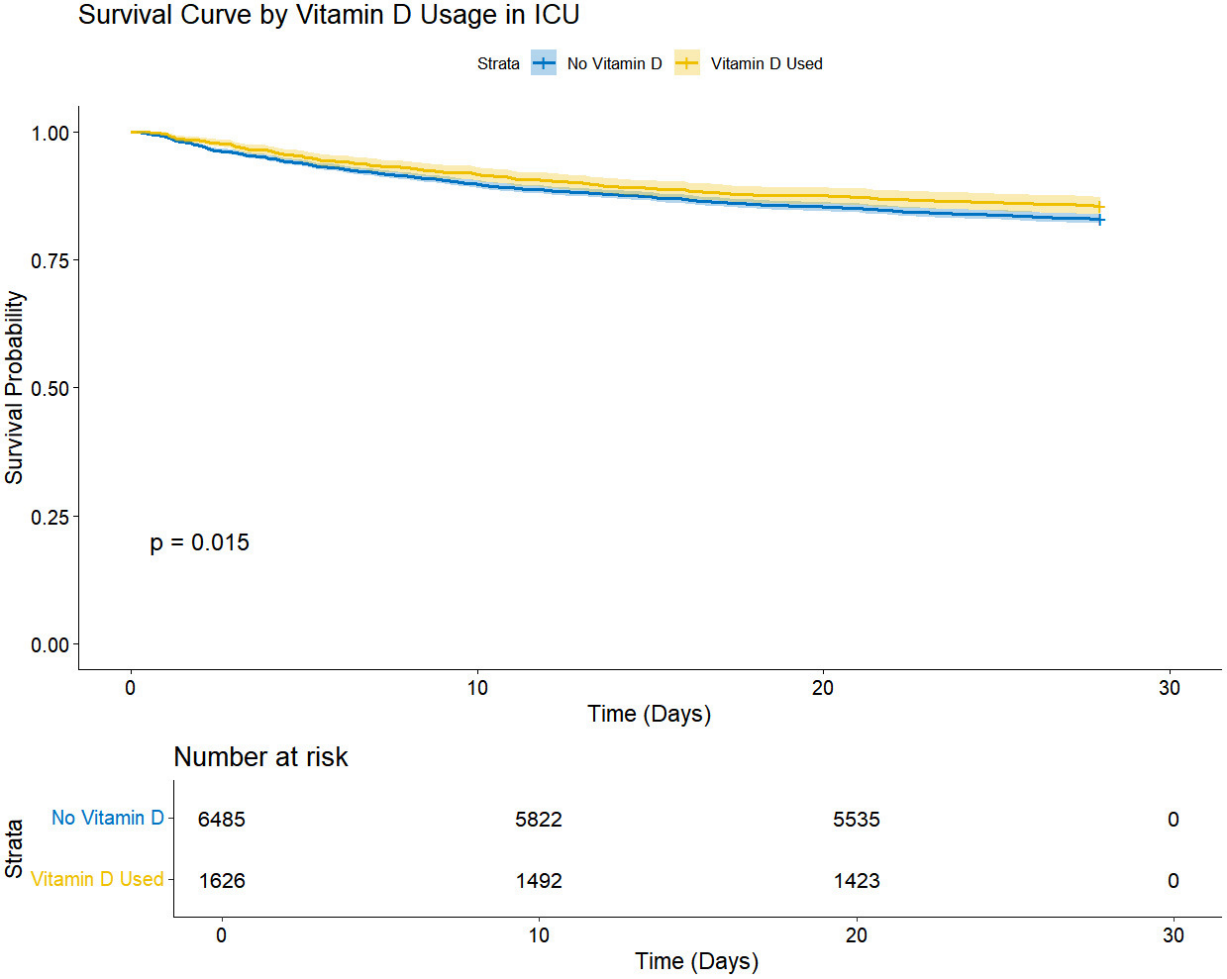
Kaplan-Meier survival curves for different patient groups. The blue line indicates patients without vitamin D supplementation, and the yellow line represents patients receiving vitamin D. The vertical axis shows cumulative survival probability, while the horizontal axis denotes follow-up time in days after ICU admission. The table below the curves displays the number of patients at risk in each group at different times. The Log-rank test revealed a statistically significant difference in survival between the two groups (*P* = 0.015).

To further assess the association between vitamin D supplementation and ICU mortality, three stepwise Cox proportional hazards models were constructed (Table 2). In the unadjusted model (Model 1), vitamin D supplementation was significantly associated with reduced mortality risk (HR: 0.840, 95% CI: 0.730–0.967, *P* = 0.015). After adjusting for demographic factors and disease severity (Model 2), the association remained significant (HR: 0.833, 95% CI: 0.724–0.959, *P* = 0.011). In the fully adjusted model (Model 3), vitamin D supplementation remained independently associated with lower 28-day mortality (HR: 0.819, 95% CI: 0.710–0.946, *P* = 0.006). The C-index increased from 0.514 to 0.770, suggesting improved model discrimination and good predictive performance.

**Table 2:** Baseline table of the study population.

Model	HR	95% CI	*P*-value
Model 1	0.840	0.730–0.967	0.015
Model 2	0.833	0.723–0.959	0.011
Model 3	0.819	0.710–0.946	0.0071

HR, hazard ratio; 95%; CI, 95% confidence interval.

### 3.3 Subgroup and sensitivity analyses

Subgroup analyses based on Model 3 were performed across sex and major comorbidities, including renal disease, diabetes, cerebrovascular disease, peripheral vascular disease, congestive heart failure, chronic pulmonary disease, and myocardial infarction (Figure 3). Vitamin D supplementation was significantly associated with reduced 28-day mortality in female patients (HR: 0.67, 95% CI: 0.54–0.82, *P* < 0.001), but not in males (HR: 1.03, 95% CI: 0.84–1.28, *P* = 0.72). Significant associations were observed in patients without renal disease (HR: 0.80, 95% CI: 0.67–0.97, *P* = 0.021), without diabetes (HR: 0.75, 95% CI: 0.62–0.90, *P* = 0.002), and without chronic pulmonary disease (HR: 0.74, 95% CI: 0.56–0.96, *P* = 0.026), whereas no significant associations were found in those with these comorbidities. Notably, greater benefits were observed in patients without congestive heart failure (HR: 0.67, 95% CI: 0.54–0.83, *P* < 0.001) and without myocardial infarction (HR: 0.78, 95% CI: 0.66–0.92, *P* = 0.004). Among patients with cerebrovascular disease, vitamin D supplementation was associated with lower mortality (HR: 0.60, 95% CI: 0.43–0.84, *P* = 0.003), while no significant association was found in those without cerebrovascular disease (*P* = 0.311).

**Figure 3:**
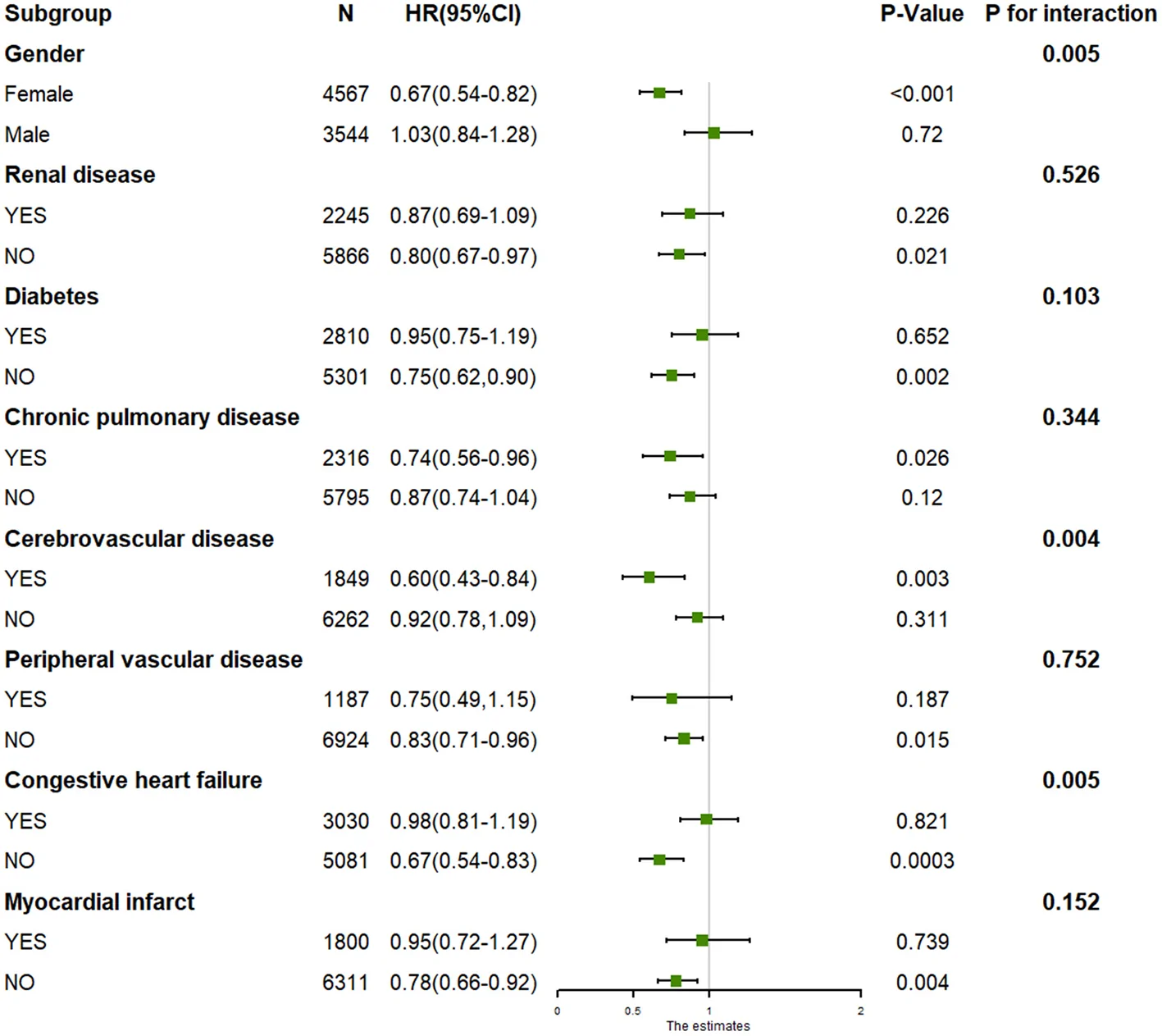
Forest plot to visualize the associations of vitamin D supplementation with 28-day ICU all-cause mortality across patient subgroups using multivariable regression analyses and meta-analysis.

Two sensitivity analyses were conducted to assess robustness. In the unmatched full cohort (*n* = 28,691), the fully adjusted model showed that vitamin D supplementation remained significantly associated with lower mortality (HR: 0.81, 95% CI: 0.71–0.92, *P* = 0.001; C-index = 0.781). The E-value for the matched cohort was 1.56 (upper 95% CI: 1.24), indicating that an unmeasured confounder would need to be associated with both exposure and outcome by a risk ratio of at least 1.56 to fully explain the observed association. Collectively, these analyses support the robustness of the findings.

### 3.4 Cumulative dose and time to first administration in relation to 28-day ICU mortality risk

In the fully adjusted restricted cubic spline analysis, using the median cumulative dose of 2,000 IU as the reference, no significant overall association was observed between cumulative vitamin D dose and 28-day ICU mortality risk (*P* overall = 0.240), nor was there evidence of a nonlinear dose–response relationship (*P* nonlinear = 0.887). The spline curve remained close to HR = 1, and the 95% confidence intervals crossed one across the entire dose range, suggesting that higher cumulative vitamin D doses were not significantly associated with changes in 28-day ICU mortality risk (Figure 4A).

**Figure 4:**
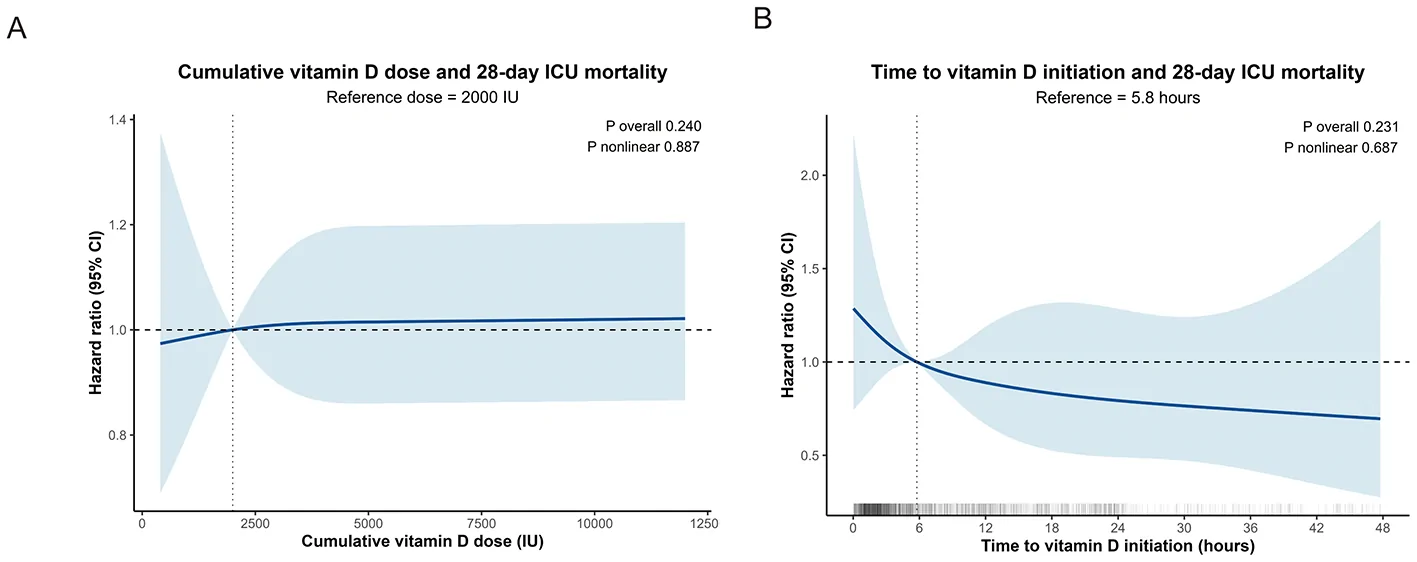
Restricted cubic spline analysis of vitamin D administration characteristics and 28-day ICU mortality risk. **(A)** Cumulative vitamin D dose, with 2,000 IU as the reference; **(B)** Time from ICU admission to first vitamin D administration, with 5.8 h as the reference. Solid lines represent fully adjusted hazard ratios, shaded areas denote 95% confidence intervals, the horizontal dashed line indicates HR = 1, and vertical dotted lines indicate the corresponding reference values.

Using a time-to-first-administration of 5.8 h after ICU admission as the reference, the time to first vitamin D administration also showed no significant overall association with 28-day ICU mortality risk (*P* overall = 0.231), nor was there evidence of a nonlinear time-response relationship (*P* nonlinear = 0.687; Figure 4B). Although the spline curve displayed a downward trend with later initiation, the 95% confidence intervals all crossed one across different time intervals. Thus, the current results do not support that earlier or later initiation of vitamin D supplementation is significantly associated with changes in mortality risk. Overall, among patients receiving vitamin D supplementation, neither cumulative dose nor time to first administration demonstrated a clear dose- or time-response relationship with 28-day ICU mortality risk.

## 4 Discussion

Currently, there is a lack of large-scale empirical evidence on the effects of vitamin D supplementation in critically ill elderly patients. Using the MIMIC-IV database, we conducted a large retrospective cohort study with propensity score matching and found that vitamin D supplementation was associated with a lower 28-day ICU mortality in older critically ill patients (HR = 0.819, 95% CI: 0.710–0.946, *P* = *0.006*). This association was consistent across subgroups, and was particularly significant among females, patients without chronic comorbidities, and those with cerebrovascular disease. The results remained robust in sensitivity analyses (E-value = 1.56). In addition, vitamin D supplementation was linked to reduced ICU and in-hospital mortality, providing new evidence for its potential clinical value in the treatment of elderly critically ill patients. These findings are consistent with previous observational studies and trends from several randomized controlled trials (RCTs), and are further supported by recent meta-analyses. In patients receiving intravenous administration, prior research suggested more pronounced benefits (*P* = 0.04) (22). Our study minimized confounding through large-sample matching and multivariable adjustment, providing more representative epidemiological evidence for the potential benefits of vitamin D supplementation.

Epidemiological studies indicate that vitamin D deficiency is highly prevalent among older adults and is closely associated with adverse outcomes such as cancer, cardiovascular events, falls, and fractures (29). Among critically ill patients, the prevalence is even higher, possibly due to prolonged bed rest, inadequate nutrition, reduced sunlight exposure, and impaired hepatic or renal function. Previous studies have shown that low 25(OH)D levels are positively correlated with disease severity scores such as SOFA and APACHE II (30). As a fat-soluble hormone precursor, vitamin D not only regulates calcium–phosphorus metabolism and maintains skeletal health but also modulates immune and metabolic gene expression through the vitamin D receptor (VDR). It suppresses excessive Th1 and Th17 inflammatory responses, thereby reducing tissue injury caused by cytokine storms (31, 32). Furthermore, the active form 1,25(OH)_2_D_3_ promotes antimicrobial peptide secretion by macrophages and epithelial cells, enhancing pathogen clearance (33). Adequate vitamin D levels also help maintain muscle strength (34), prevent muscle atrophy and delayed recovery during prolonged immobility, and contribute to neuroprotection and cardiovascular homeostasis (35, 36). Collectively, these mechanisms suggest that vitamin D may improve systemic stability and organ function in elderly critically ill patients through immune regulation, metabolic balance, and neuroprotective pathways, ultimately reducing mortality risk.

Our study further suggests that the benefits of vitamin D supplementation may be more pronounced in specific subgroups. First, supplementation was associated with lower mortality in patients with cerebrovascular disease but not in those without. This may be related to its ability to reduce neuroinflammation, preserve the integrity of the blood–brain barrier, and promote neuronal repair (37–39). Mechanistically, 1,25(OH)_2_D_3_ may inhibit NF-κB/NLRP3 signaling via VDR, thereby attenuating inflammatory cascades and protecting the central nervous system, which could lower the risk of delirium and neurological dysfunction (40, 41). Second, sex differences may influence the therapeutic effect. We observed a significant reduction in mortality among female patients, but not among males. A 2023 epidemiological study reported that estradiol levels modulate the interaction between vitamin D and low skeletal muscle mass in middle-aged and elderly women, suggesting that hormonal status may influence vitamin D's biological effects (42). Katia Danza et al. proposed that the ERR–VDR axis may weaken VDR signaling while enhancing estrogen-related pathways (43), implying that estrogen may enhance the responsiveness to vitamin D supplementation in women. Finally, the presence of chronic diseases may attenuate the benefit of supplementation. In patients without kidney disease, diabetes, or chronic lung disease, vitamin D supplementation was significantly associated with reduced mortality; however, this association was not observed in those with these comorbidities. This difference may be due to chronic disease–related alterations in vitamin D metabolism and signaling (44–46). Studies have shown that chronic kidney disease reduces 1α-hydroxylase activity, while diabetes and chronic lung disease are often accompanied by persistent inflammation and metabolic disturbances, which may blunt vitamin D's physiological effects (47–49). Taken together, vitamin D represents a safe, inexpensive, and feasible intervention. Early identification of deficiency and timely supplementation may improve outcomes in elderly critically ill patients. Future studies should consider incorporating vitamin D status assessment into nutritional evaluation systems for critically ill populations and explore the optimal dose, route, and timing of intervention to establish evidence-based clinical guidelines.

Our observational findings (HR 0.819) align with some meta-analyses suggesting a mortality benefit (RR 0.78–0.83) (21, 22), but differ from several RCT-level meta-analyses that reported no significant mortality reduction (50, 51). This discrepancy may be attributable to several factors. First, RCTs often restrict enrollment to patients with documented vitamin D deficiency (52), whereas we could not ascertain baseline vitamin D status, potentially diluting the observed effect if benefit is predominantly confined to severely deficient patients (53). Second, the supplementation regimens differ substantially: our median cumulative dose was only 2,000 IU enterally, while RCTs typically employ high-dose protocols (e.g., 5,40,000 IU loading dose) (50), and parenteral administration may confer greater efficacy (22). Third, observational studies are subject to residual confounding and confounding by indication, as clinicians may selectively supplement patients based on unmeasured factors linked to prognosis (53). Fourth, recent evidence suggests that mechanically ventilated patients may derive more benefit (21), yet the supplementation group in our cohort had a lower proportion of invasive ventilation (21.5% vs. 35.9%). Collectively, these differences highlight the need for large-scale, well-designed RCTs with baseline vitamin D stratification to definitively establish the efficacy of vitamin D supplementation in elderly critically ill patients.

Compared with previous studies, our work has several strengths. First, the large sample size and comprehensive adjustment for multiple covariates enhance the reliability and generalizability of our findings. Second, this study addresses key limitations of earlier research, which often focused on general ICU populations or specific disease groups. By targeting a large, well-matched elderly cohort, we provided more specific evidence for this high-risk population. Finally, the inclusion of extensive subgroup and sensitivity analyses allowed us to identify potential heterogeneity by sex and comorbid status, offering insights for future precision supplementation strategies and clinical decision-making.

Nevertheless, this study has several limitations. As a retrospective analysis, residual confounding cannot be fully excluded despite propensity score matching and multivariate adjustment. Residual confounding by indication remains a concern because the clinical indications for vitamin D supplementation were not available in the database, although we adjusted for measured covariates via propensity score matching and multivariable regression. Immortal time bias was addressed by defining a fixed 48-hour exposure window; however, unmeasured confounders such as baseline vitamin D status cannot be ruled out, and causality should not be inferred. In addition, Given the multiple comparisons performed in the subgroup analyses, the possibility of false-positive findings cannot be excluded. Although interaction tests were conducted, these results are exploratory and should be confirmed by further studies before drawing firm conclusions.

## 5 Conclusion

In this large retrospective cohort study, vitamin D supplementation within 48 hours of ICU admission was independently associated with a significant reduction in 28-day all-cause mortality in elderly critically ill patients. The association was more pronounced in female patients, those without chronic comorbidities, and patients with cerebrovascular disease. These findings suggest that vitamin D supplementation may be associated with improved outcomes, but causality requires confirmation in prospective randomized trials.

## Data Availability

The original contributions presented in the study are included in the article/Supplementary material, further inquiries can be directed to the corresponding authors.
