# Supplementary material for: Association between vitamin D supplementation and mortality in elderly critically ill patients: a retrospective propensity score–matched cohort study
Source: Front Nutr. 2026 Sep 17;13:1932001. doi: 10.3389/fnut.2026.1932001 (PMC13626891; doi:10.3389/fnut.2026.1932001)
Supplement: Supplementary file 1 [file Table_1.doc]

**Supplement table 1.** Missing proportion

| Variable | Missing Count | Missing Percentage (%) |
| --- | --- | --- |
| temperature | 1027 | 3.58 |
| rbc | 550 | 1.92 |
| wbc | 546 | 1.9 |
| plt | 541 | 1.89 |
| bun | 424 | 1.48 |
| HCO3 | 423 | 1.47 |
| cr | 417 | 1.45 |
| K | 395 | 1.38 |
| Na | 391 | 1.36 |
| glucose | 154 | 0.54 |
| resp_rate | 73 | 0.25 |
| spo2 | 39 | 0.14 |
| sbp | 37 | 0.13 |
| dbp | 37 | 0.13 |
| sofa | 35 | 0.12 |
| heart_rate | 35 | 0.12 |
| sapsii | 0 | 0.00 |
