# Supplementary material for: Association between vitamin D supplementation and mortality in elderly critically ill patients: a retrospective propensity score–matched cohort study
Source: Front Nutr. 2026 Sep 17;13:1932001. doi: 10.3389/fnut.2026.1932001 (PMC13626891; doi:10.3389/fnut.2026.1932001)
Supplement: Supplementary file 2 [file Table_2.doc]

**Supplement table 2.** GVIF Table Before Matching

|  | GVIF |
| --- | --- |
| gender | 1.103 |
| age | 1.465 |
| charlson_comorbidity_index | 4.034 |
| sofa | 1.755 |
| sapsii | 1.951 |
| heart_rate | 1.321 |
| sbp | 1.593 |
| dbp | 1.612 |
| resp_rate | 1.232 |
| temperature | 1.154 |
| spo2 | 1.14 |
| glucose | 1.001 |
| rbc | 1.256 |
| wbc | 1.086 |
| plt | 1.176 |
| Na | 1.117 |
| K | 1.18 |
| HCO3 | 1.242 |
| bun | 2.26 |
| cr | 2.233 |
| severe_liver_disease | 1.181 |
| malignant_cancer | 1.794 |
| renal_disease | 1.989 |
| diabetes | 1.313 |
| chronic_pulmonary_disease | 1.198 |
| cerebrovascular_disease | 1.317 |
| peripheral_vascular_disease | 1.103 |
| congestive_heart_failure | 1.383 |
| myocardial_infarct | 1.191 |
| invasive_ventilator_flag | 1.393 |
| crrt_flag | 1.098 |
| dopamine_used | 1.05 |
| dobutamine_used | 1.033 |
| epinephrine_used | 1.176 |
| norepinephrine_used | 1.342 |
